# Executive and attentional functioning interventions in preterm children: a systematic review

**DOI:** 10.1093/jpepsy/jsae068

**Published:** 2024-08-26

**Authors:** Yara Maalouf, Sarah Provost, Isabelle Gaudet, Philippe Dodin, Natacha Paquette, Anne Gallagher

**Affiliations:** Neurodevelopmental Optical Imaging Laboratory (LIONlab), Research Center, CHU Sainte-Justine University Hospital Center, Montréal, QC, Canada; Department of Psychology, Université de Montréal, Montréal, QC, Canada; Neurodevelopmental Optical Imaging Laboratory (LIONlab), Research Center, CHU Sainte-Justine University Hospital Center, Montréal, QC, Canada; Department of Psychology, Université de Montréal, Montréal, QC, Canada; Neurodevelopmental Optical Imaging Laboratory (LIONlab), Research Center, CHU Sainte-Justine University Hospital Center, Montréal, QC, Canada; Department of Health Sciences, Université du Québec à Chicoutimi, Chicoutimi, QC, Canada; Library, CHU Sainte-Justine University Hospital Center, Montréal, QC, Canada; Neurodevelopmental Optical Imaging Laboratory (LIONlab), Research Center, CHU Sainte-Justine University Hospital Center, Montréal, QC, Canada; Neurodevelopmental Optical Imaging Laboratory (LIONlab), Research Center, CHU Sainte-Justine University Hospital Center, Montréal, QC, Canada; Department of Psychology, Université de Montréal, Montréal, QC, Canada

**Keywords:** prematurity, neuropsychology, intervention outcome, systematic review

## Abstract

**Objective:**

This systematic review, performed in accordance with the PRISMA guidelines, seeks to summarize the interventions that have been developed in order to improve executive functioning and attention in children born prematurely.

**Methods:**

The PICOS framework helped guide the structure and relevant terms selected for the study. Electronic systematic searches of the databases PubMed (NLM), Ovid Medline, Ovid All EBM Reviews, Ovid Embase, and Ovid PsycINFO were completed in March 2022. This review focuses on interventions that target attention and executive functioning in prematurely born children between birth and 12 years old, with outcome measures assessed between 3 and 12 years old, even if the age range in the study can exceed our own parameters. Data extraction included sample characteristics, country of recruitment, type of intervention, description of the intervention group and control group, outcome measures, and overall results. An assessment of the quality of methodology of studies was performed through an adaptation of the Downs and Black checklist for both randomized and nonrandomized studies in healthcare interventions. An assessment of the risk of bias was also presented using the Cochrane risk of bias tool for randomized trials 2.0.

**Results:**

A total of 517 premature children received an intervention at some point between birth and early adolescence. Eleven different interventions were assessed in 17 studies, with rating of the quality of methodology and outcomes ranging from lower quality studies (44% quality rating) to robust studies (96% quality rating) in terms of reporting standards, external and internal validity, and power. Five of those studies focused on interventions administered in the neonatal intensive care unit or shortly postdischarge (e.g., the Mother–Infant Transaction Program and the Newborn Individualized Developmental Care and Assessment Program, documented in two articles each [11%] or the Infant Behavioral Assessment and Intervention Program assessed in one study [about 5%]), while 12 articles reported on interventions administered between the ages of 1.5–12 years old [mostly computerized cognitive training programs such as Cogmed (23%) and BrainGame Brian (17%)]. Of the 17 articles examined, 12 (70%) showed positive short-term outcomes postintervention and 3 (17%) demonstrated positive long-term results with small to large effect sizes (0.23–2.3). Among included studies, 50% showed an overall high risk of bias, 21.4% showed some concerns, and 28.6% were low risk of bias.

**Conclusions:**

Due to the heterogeneity of the programs reviewed, the presented findings should be interpreted as descriptive results. A careful and individualized selection from the various available interventions should be made based on the target population (i.e., age at intervention administration and outcome testing) before implementing these program protocols in clinical settings.

## Introduction

According to the World Health Organization (2023) report, an estimated 152 million babies were born prematurely (under 37 weeks of gestation) between 2010 and 2020. Worldwide, this represents one premature birth every 2 s, accounting for almost 10% of all live births. A classification of premature birth is documented according to gestational age (GA): moderate or late preterm: 32–36 weeks, very preterm: 28–32 weeks, and extremely preterm <28 weeks. Despite global efforts and research collaborations, prematurity rates have remained virtually stable in the last 10 years, and prematurity continues to be the leading cause of death in children under 5 years old. Fortunately, about 85% of premature births are classified as moderately preterm, not necessitating a prolonged hospital stay. Nonetheless, the developmental footprint of prematurity remains immense on children and their families, and persists throughout their lives (World Health Organization, 2023).

### Brain development in premature children

The gestational period is a crucial time for brain development, rendering it a very vulnerable phase. In neurotypical development, neurulation, neuronal proliferation and neural migration begin at 3 weeks of GA and continue until birth. During the second and third trimesters of gestation, the processes of apoptosis, synaptogenesis, and myelination occur and continue after birth (Tau and Peterson, 2010). In infants born prematurely, it is well documented that brain tissue volumes, specifically cortical and subcortical gray matter and the cerebellum, are lower than that of healthy term controls at term-equivalent age, while more cerebrospinal fluid is present. This reduction in brain tissue volumes and increase in fluid in the preterm brain is positively associated with GA at birth (Keunen et al., 2012). A reduction of or injury of white matter has also been reported in premature infants and is equally dependent on GA (Ment et al., 2009; Molnár and Rutherford, 2013; Schneider and Miller, 2019). The effect of prematurity on brain development persists well into adolescence, as preterm teens show less cortical thickness compared to their term-born adolescent peers (Nagy et al., 2011). Neurotransmitter systems such as the GABAergic system, hypothesized to play an important role in several neurodevelopmental disorders, are also altered after a preterm birth, and show a maturation delay in the prefrontal cortex compared to full-term infants (Lacaille et al., 2021).

### Neurodevelopment in premature children

Prematurity has been shown to be a risk factor for neurodevelopmental disorders due to several factors including immature brain development and white matter injuries (Cainelli et al., 2020; Fleiss et al., 2020). Indeed, children born preterm have a higher risk (two- to threefold) for attention-deficit/hyperactivity disorder (ADHD) than their term-born peers, and this risk increases with lower GA and poorer fetal growth (Montagna et al., 2020; Sucksdorff et al., 2015). Children born prematurely exhibit more cognitive and fine and gross motor deficits (Allen, 2008; Jarjour, 2015). Notably, premature populations ranging from childhood to adulthood are at increased risk of developing problems in higher-level cognitive functions, such as attention and executive functioning (Breeman et al., 2016; Taylor and Clark, 2016). These neurodevelopmental sequelae often lead to challenges in social and daily life, as well as in the academic sphere once these children have reached school age, including lower scores in mathematics, reading, spelling, and writing, and increased risk of learning disabilities (Keller-Margulis et al., 2011). Given the vast impact of executive and attentional difficulties on overall functioning in the preterm population, these two cognitive functions serve as an ideal target for the development of diverse interventions such as cognitive training. Such interventions are geared toward improving these functions as well as the quality of life associated with their optimal functioning. Furthermore, the need to implement early interventions is evident considering the neuroplasticity of the young brain (Weyandt et al., 2020). Indeed, early childhood is a critical period for brain development characterized by a highly adaptable brain, with an enhanced capacity and ease to form and rearrange neural connections in response to diverse interventions. Programs implemented in this developmental period are therefore ideal to leverage this neuroplasticity, mitigate global developmental delays, and enhance long-term cognitive development (Cramer et al., 2011; Weyandt et al., 2020). While some of these interventions have been the focus of reviews in clinical populations such as ADHD (Westwood et al., 2023), the effect of these interventions on attention and executive functions has not been studied yet in the preterm population.

### Executive functions and attention

Neurodevelopmental disorders have been linked to executive and attentional deficits at an early age (Soleimani et al., 2014; Sun and Buys, 2012, Van de Weijer-Bergsma et al., 2008). Executive functions are a subset of high-level cognitive functions that enable adaptation to new situations by allowing problem-solving, orienting focus, and decision-making (Diamond, 2020). Some models have identified the three core components of executive functions to be *working memory*, the ability to hold in mind, update, and manipulate information; *inhibitory control*, the ability to inhibit or override an urge, or an entrenched habit or impulse, in order to do what is more appropriate or required; and *cognitive flexibility* or shifting, the ability to shift tasks, perspectives, and focus, and to adjust or adapt to changes (Diamond, 2011, 2020). In those models, these three fundamental skills serve as a crucial basis for the emergence of more advanced executive functions (e.g., problem-solving, reasoning, planning, and organizing) as well as other cognitive processes (e.g., sustained and divided attention; Devine et al., 2019; Diamond, 2011, 2020). In typically developing and premature children, early executive functions predict school readiness and academic achievement in math and reading throughout primary and secondary education (Christopher et al., 2012; Cortés Pascual et al., 2019; Costa et al., 2017). In both healthy and preterm populations, early executive functions are also associated with behavior, social adjustment and competence, and quality of life in later childhood, adolescence, and adulthood (Diamond, 2011, 2020; Wolfe et al., 2015). Executive functions are mostly supported by the prefrontal cortex and its connecting areas and play a critical role in overall brain functioning (Miller and Wallis, 2009). Tremendous development of the prefrontal area and executive functions occurs in the preschool period (3–5 years old); however, the executive processes solidify and are more evident in school-aged and preadolescent children (6–12 years old; Diamond, 2020). Considering the important role of executive functioning on overall cognitive development and quality of life, a myriad of interventions have been developed to improve this function. Most interventions involve cognitive training of one or several core components of executive functions as well as physical activity programs (Diamond and Ling, 2016).

Attentional skills are closely related to executive functions (Diamond, 2020). In fact, concordant with what is seen in healthy term-born children, executive functioning seems to play a role in attentional difficulties, highlighting the interaction between these two high-order processes in premature children (Eves et al., 2020). Three distinct networks of attention have been described: an *alerting* network, related to vigilance which allows us to exit the resting state and respond to a new stimulus by deploying our attentional resources; an *orienting* network, allowing us to direct our focus toward a specific cue; and an *executive control* network responsible for prioritizing incoming stimuli and selecting only relevant ones (Markett et al., 2022; Petersen and Posner, 2012). Although the brain networks recruited by these three mechanisms are distinct, research shows that they overlap, primarily in the dorso-fronto-parietal and mid-cingulo-insular brain networks (Markett et al., 2022; Petersen and Posner, 2012). In typically developing infants, a rudimentary form of attention can be observed as early as 6 months old (Posner et al., 2020). The development of attentional skills also overlaps with that of working memory and joint attention (Mundy and Newell, 2007; Reynolds and Romano, 2016). Several interventions aiming to improve attention have been identified. According to certain authors, they can be classified into two training strategies. Network training focuses on improving the efficiency of brain networks involved through the practice of a specific cognitive task (e.g., working memory training protocols), whereas state training does not involve the practice of a single task but rather creating a brain state that may influence one’s capacity for attention (e.g., mindfulness-based techniques; Posner et al., 2015).

### Objective

In order to overcome or minimize the neurodevelopmental consequences of preterm birth, several interventions have been developed to improve executive functioning and attention in preschool-aged, school-aged, and preadolescent children born prematurely. These interventions tend to diverge from a methodological point of view, are administered at different developmental stages, and yield inconsistent results. The objective of this review is to summarize interventions that have been developed from birth to preadolescence to improve executive functioning and attention in children aged 3–12 years old born prematurely. Specifically, this systematic review seeks to clarify several questions including (1) the benefits and efficacy of the interventions on the executive and attentional functioning of premature children; (2) the noteworthy characteristics of the interventions; and (3) the relationship between the age of administration of the intervention and the efficacy of the program.

## Method

### Search strategy

This systematic review was conducted by following the guidelines of the preferred reporting items for systematic reviews and meta-analyses (PRISMA) method (Moher et al., 2009). The final PRISMA checklist is available as online supplementary file S1. An initial review of relevant search terms and keywords were selected by some of the authors (Y.M., S.P., I.G., N.P., and A.G.) following the PICOS framework structure. The keywords were refined and elaborated by a librarian from the Sainte-Justine University Health Center (P.D.). Electronic systematic searches of the PubMed (NLM), Ovid Medline, Ovid All EBM Reviews, Ovid Embase, and Ovid PsycINFO databases were completed in March 2022 by a librarian (P.D.) with expertise in literature searches. All articles published up to and including March 2022 were incorporated into the search. The search strategy was modified slightly according to the criteria of the different databases. Key terms included synonyms, variations, and terminologies related to preschool, school-aged and preadolescent children, interventions and programs, executive functions and/or attention, and prematurity. A full description of the search strategy used for each database is available in Appendix A. The results were exported by the librarian (P.D.) to an EndNote X9 library. The study was not registered.

### Selection criteria

**Figure 1.**
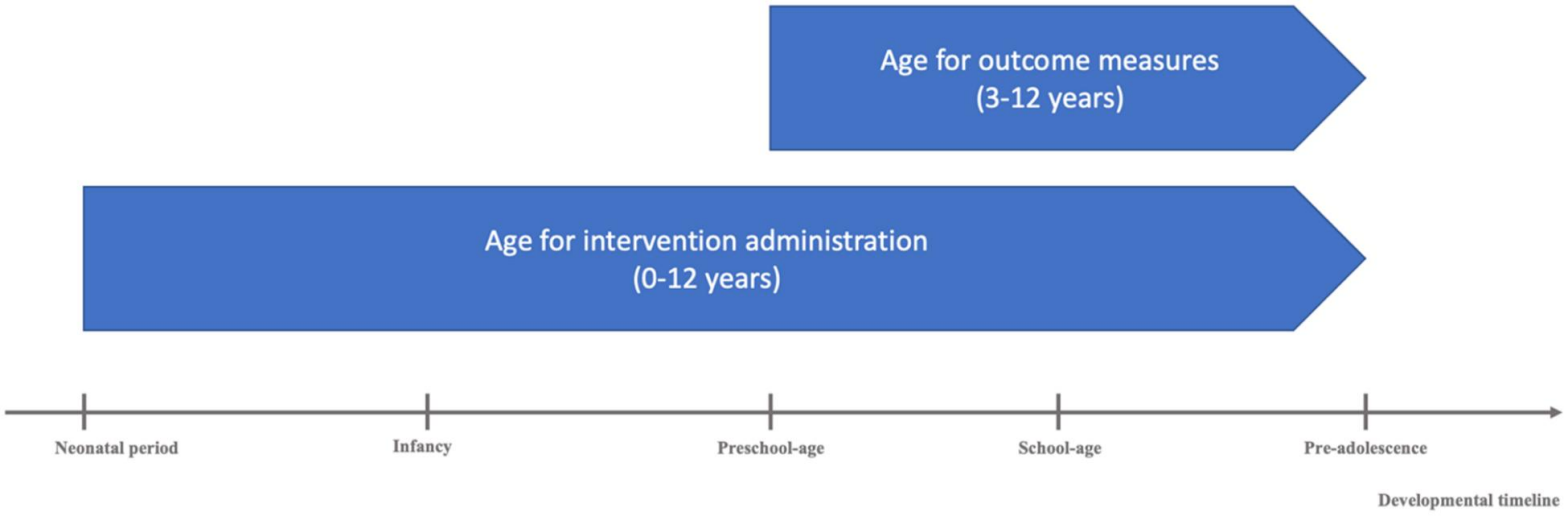
Summary of age selection for intervention administration and outcomes measures.

The inclusion criteria were as follows: (1) preschool, school-aged children, and preadolescents aged between 3 and 12 years old at the time of the outcome measures, even if the age range in the study exceeded our own parameters, although the intervention could have been administered earlier in development (i.e., infants and toddlers below the age of 3 years; see Figure 1 for summary of age inclusion); (2) studies that assessed the efficacy of an intervention program; (3) articles that included at least one performance-based (objective) and/or daily-life (subjective) outcome measure of executive functions and/or attention; (4) children born prematurely (GA <37 weeks), including children born extremely prematurely, very prematurely, and/or moderate to late premature children. The age study parameters were set from 3 to 12 years old for outcome measures, as the adolescence and adulthood periods differ from the childhood period in relation to therapeutic modalities, subsequent impact of intervention on cognitive functioning, underlying mechanisms of potential changes following intervention, and cerebral changes (Everts et al., 2019; Kelly et al., 2024).

Empirical studies published in French or English in peer-reviewed journals were included. This review was not limited to randomized controlled trials with an active or passive control group and included studies using no comparison groups in their design (i.e., single-subject design could be included). Both longitudinal and cross-sectional studies were included in this review. For longitudinal studies, only the outcome measures assessed between 3 and 12 years old (selection criteria 1) were discussed. Gray literature, case studies, and case series were excluded from this review.

### Data extraction

The librarian (P.D.) removed all duplicates from the list of citations. The first selection, including the review of all titles and abstracts, was done by the first author (Y.M.). The second selection was done independently by the first two investigators (Y.M. and S.P.) and used the full text in order to apply selection criteria. A third author (I.G.) was consulted when discrepancies were observed and helped determine eligibility of the study through discussion with the two other authors until a consensus was reached. Data were then extracted from the relevant articles by the first author (Y.M.) and summarized in a spreadsheet. Data extraction included sample characteristics (age, number of participants, sex, ethnicity, GA), country of recruitment, type of intervention, description of the intervention (duration and intensity of sessions), description of control groups (active, passive or none), outcome measures (executive functions, attention, or others) and overall results, including effect sizes for studies where this information was available. Effect sizes were categorized as small (*d* = 0.2), medium (*d* = 0.5), or large (*d* ≥ 0.8) according to Cohen’s guidelines (1992). Missing data were indicated as “not available”. No attempts to contact the original study authors were initiated. Due to the heterogeneity of studies included, it was necessary to conduct a systematic review instead of a meta-analysis as our raw data could not be pooled.

### Quality assessment and risk of bias assessment

An assessment of the quality of the methodology and outcome of empirical studies presented in this review was performed through an adaptation of the Downs and Black (1998) checklist for both randomized and nonrandomized studies of healthcare interventions, which helped us assess the certainty in the body of evidence of the outcomes obtained. The checklist encompasses questions related to five domains: (1) reporting: which assesses the amount of information reported in a study (e.g., characteristics of participants and lost to follow-up participants, intervention details, probability values); (2) external validity, which addresses the generalizability factor of the outcomes (e.g., how representative the facility, staff, and place where the intervention is received); (3) internal validity bias, which addresses the bias in the measurement of the intervention and the outcome (e.g., mention of blind assessors or data dredging); (4) internal validity: confounding (selection bias), which refers to the bias in selecting specific study participants (e.g., randomization and concealment information), (5) power, which determines if findings could be due to chance (i.e., if the study has enough power to detect a clinically important effect). The quality was independently assessed by the first author (Y.M.) and co-authors (S.P. and I.G.) for three studies (18% of studies) to ensure interrater agreement. This checklist is commonly used in other systematic reviews and meta-analyses to evaluate healthcare intervention (Chavez-Arana et al., 2018; Edwards et al., 2022; Joplin et al., 2018). Details of the modified scale are available in Appendix B. The total maximum score obtained was 27, which was then computed into a percentage. The indicator “unable to determine” was selected for missing data in manuscripts. The scores obtained for the checklist did not serve as a criterion for inclusion in the study (i.e., no study was eliminated because the score was too low) but helped nuance the results presented below. Furthermore, an assessment of the risk of bias was presented using the Cochrane risk of bias tool for randomized trials 2.0 (Higgins et al., 2019). The tool assesses five risks of bias domains including: bias arising from the randomization process, bias due to the deviations from the intended interventions, bias due to missing outcome data, bias in measurement of the outcome and bias in selection of reported result. An overall bias assessment was also derived. The risks were categorized as low, some concerns or high. The aim of the review was to assess the effect of assignment to intervention (i.e., intention-to-treat effect). The risk of bias was assessed by the first author (Y.M.) for all studies and supporting author (S.P.) for 8 (47% of studies) randomly selected studies or more methodologically complex studies to ensure partial interrater agreement.

## Results

**Figure 2.**
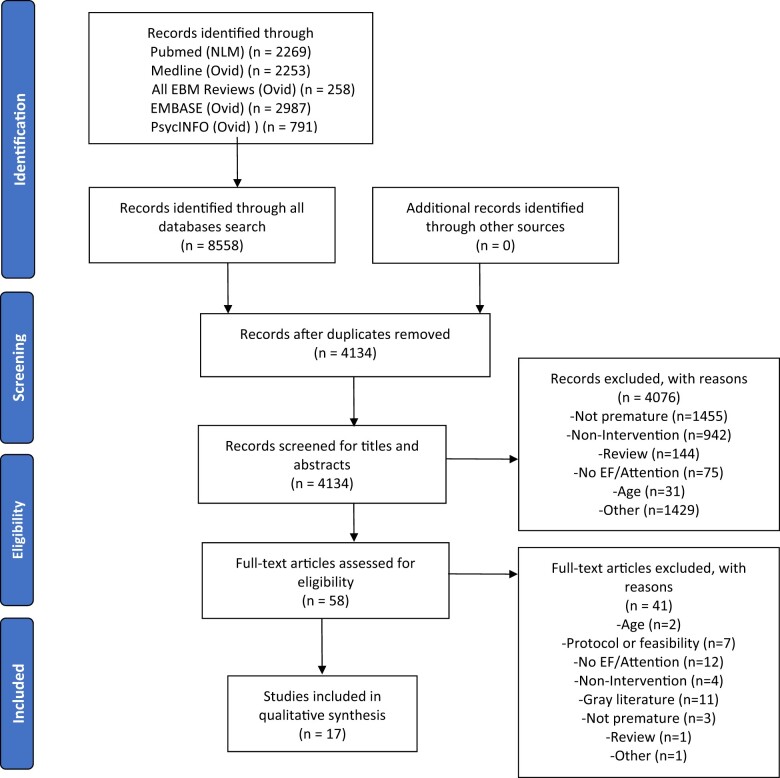
PRISMA workflow diagram of study selection.

The PRISMA workflow diagram for study selection is presented in Figure 2. After removing duplicates, a total of 4134 records were identified. Of these, 4076 records were excluded based on the criteria after the first selection for (1) being systematic or literature reviews; (2) not evaluating the efficacy of an intervention; (3) not having children born prematurely as a population of interest and had instead children with cerebral palsy, autism, etc.; (4) not measuring any outcomes related to executive functions or attention; (5) being aimed at adolescents aged over 12 years old or at adults; (6) not being related to the objective of this systematic review. A total of 41 articles out of 58 were excluded after screening the full text because they were primarily protocols, conference abstracts, graduate theses or did not include an executive functioning or attention measure (see online supplementary Table S1). Discrepancies in eligibility of records were noted for four articles and were resolved through deliberation with the third author (93.1% interrater agreement). Ultimately, 17 articles met the selection criteria and assessed attention or executive functions in children aged between 3 and 12 years old who had previously undergone an intervention several weeks to years before the evaluation. They were published between 2004 and 2021 in 10 different Western countries.

### Overview of results

A summary of the results is presented in Table 1 for studies that examined the effect of interventions received during infancy (under 1 year old) and Table 2 for studies that reviewed programs implemented for toddlers and preschoolers (1.5–5 years) and school-aged and preadolescent children (6–14 years old). The interventions received under 1 year old were separated in the results given that they were administered in the neonatal intensive care unit (NICU) and/or shortly postdischarge and have very different protocols from the programs implemented over 1 year old. The outcome results for the interventions received during infancy, toddlerhood, preschool period, school-age period, and preadolescence were all measured sometime between 3 and 12 years old, although some interventions occurred years before the outcomes were measured. It is noteworthy that one article was included in our systematic review despite the age range for children being 10–14 years at the time of outcome measures (Siffredi et al., 2021), considering that a proportion of children were between the ages of 10 and 12 years old at the time of outcome measures. Similarly, another article was included despite the participants being between 1.5 and 5 years old at the time of outcome measures (Bagner et al., 2010), considering that a proportion of children were between the ages of 3 and 5 years old at the time of outcome measures.

**Table 1.** Summary of results of interventions during neonatal and infancy period (under 1-year old at the time of intervention).

First author, year [country]		Participants’ description			Intervention characteristics		Outcome measures		Results, *p*-value (*p*), effect size (*d*)	Quality score of methodology
	Control group	*N* (*F*%)	Ethnicity (Black [B], White [W], Hispanic [H], other [O], etc.)	GA, mean (*SD* or range)	Age/place of intervention	Type and duration of intervention	Age at outcome measures	EF or attention variables (instruments, subtest)		
Milgrom, 2019 [Australia]	Standard care for preterm infants	Intervention: 60 (50%) Control: 63 (52%)	N/A	Intervention: 27.4 (1.5) Control: 27.8 (1.7)	In NICU + 1 visit postdischarge	Mother–infant (MITP-type), 10 sessions (60 min/session), over 13 weeks	2 and 4.5 years	Inhibition (NEPSY-II) Attention (CBCL)	N.S. on all outcome measures (*d* = N/A)	88%
Landsem, 2015 [Norway]	Standard care for preterm infants and standard care for infants born at term	Intervention: 72 (46%) Control: 74 (46%) for preterm and 75 (46%) for born at term	N/A	Intervention: 30.2 (3.1) Control: 29.9 (3.5) for preterm and 39.3 (1.3) for born at term	In NICU + 4 visits postdischarge	Mother–infant (MITP), 11 sessions (60 min/session), over 19 weeks	2, 3, 5, 7, and 9 years	Attention (CBCL) Attention (TRF)	Fewer attention problems (*p* ≤ .03, *d* = 0.48), fewer thought problems (*p* ≤ .05, *d* = [0.40–0.52]), fewer difficulties (*p* = .02, *d* = 0.48) and better adaptation (*p* ≤ .06, *d* = [0.45–0.62]) to school as reported by parents and teachers at age 7 and 9 years old	88%
McAnulty, 2013 [USA]	Standard care for preterm infants	Intervention: 9 (55%) Control: 14 (35%)	Intervention: B: 1, W: 7, H: 0, O: 0 Control: C: 10, B: 1, H: 1, O: 2	Between 29 and 33 weeks	In NICU	NIDCAP, daily sessions (duration not specified) until 2 weeks corrected age	9 years	Planning and organizing (ROCFT)	Significantly better accuracy score on copy condition (*p* = .05, d = N/A) and overall better mean scores in the intervention group	92%
Westrup, 2004 [Sweden]	Standard care for preterm infants	Intervention: 11 (23%) Control: 15, (42%)	N/A	Intervention: 27.6 (24.0–28.7) Control: 26.1 (23.9–30.3)	In NICU	NIDCAP, weekly sessions (duration not specified) starting within 3 days of birth until 36 weeks postconception	6 years	Attention and hyperactivity (NEPSY)	Less children in the intervention group had significant behavioral deficits (*p* = .04, d = N/A)	81%
Verkerk, 2012 [The Netherlands]	Standard care for preterm infants and standard care for infants born at term	Intervention: 76 (43%) Control: 75 (57%) for preterm and 41 (48%) for born at term	N/A	Intervention: 29.6 (2.2) Control: 30.0 (2.1) for preterm and n.a. for born at term	Hospital + 6–8 sessions postdischarge	Parent–infant (IBAIP), 7–9 sessions (60 min/session), until 6 months corrected age	3.6 years	Attention (CBCL) Attention (VAT) Inhibition, working memory, planning and organizing, emotional control, and shift (BRIEF-P) Inhibition (Gift delay task)	N.S. on all outcome measures (*d* = N/A). Positive significant interaction effect on attention and EF for children with GA < 28 weeks	70%

*Note.* BRIEF-P = behavior rating inventory of executive function, preschool version; CBCL = Child Behavior Checklist; F = female; IBAIP = The Infant Behavioral Assessment and Intervention Program; MITP = mother–infant transaction program; n.a. = not available; NEPSY = a developmental neuropsychological assessment; NIDCAP = The Newborn Individualized Developmental Care and Assessment Program; n.s. = nonsignificant; N/A = not applicable/available; ROCFT = Rey-Osterrieth Complex Figure Test; TRF = teachers report form; VAT = visual attention task.

**Table 2.** Summary of results of interventions during toddlerhood, preschool and school-age, and preadolescence (1.5–14 years old).

Author, year [Country]		Participants’ description			Intervention characteristics		Outcome measures		Results, *p*-value (*p*), effect size (*d*)	Quality score of methodology (in %)
	Control group	*N* (*F*%)	Ethnicity (Black [B], White [W], Hispanic [H], Asian [A], Other [O], etc.)	GA, mean (SD or range)	Age at intervention	Type and duration of intervention	Age at outcome measures	EF or attention variables (instruments)		
Bagner, 2010 [USA]	Waitlist for preterm children	Intervention: 14 (29%) Control: 14 (29%)	B: 4%, W: 82%, H: 21%, A: 4%, O: 10%	Intervention: 28 (23–35) Control: 28.5 (25–34)	Between 1.5 and 5 years (mean age: 3.3 years)	Parent–child (PCIT), weekly sessions (60 min/session) until the parent demonstrate mastery of interaction skill (average of 13 sessions)	Between 1.5 and 5 years (mean age: 3.3 years)	Disruptive behavior (ECBI) Attention (CBCL)	Significantly less attention problems (*p* ≤ .011, d = [1.1–2.3]) and fewer disruptive and problematic behavior (*p* ≤ .011, d = [1.4–2.3]) in the intervention group. Results maintained 4 months postintervention	92%
Slevin, 2020 [Ireland]	No intervention for preterm children	Intervention: 9 (44%) Control: 9 (55%)	N/A	Intervention: 26.5 (N/A) Control: 26.6 (N/A)	Between 3 and 4 years (mean age: 3.1 years)	Child, therapeutic listening, 5×/week (duration not specified) for 6 months	Between 3 and 4 years (mean age: 3.1 years)	Attention (Reynell Attention Scale)	Attention level improved by two levels for seven children and one level for two children out of nine in the intervention group (*p* = N/A, *d* = N/A)	44%
García-Bermúdez, 2019 [Spain]	General curricular intervention for preterm children	Intervention: 36 (63%) Control: 32 (46%)	N/A	Intervention: 35.18 (1.51) Control: 34.73 (1.84)	Between 4 and 5 years (mean age: 5 years)	Child, PEFEN program, weekly sessions (150 min/session) for 3 months (12 sessions in total)	Between 4 and 5 years (mean age: 5 years)	Working memory (BENCI) Inhibition, working memory, planning and organizing, emotional control, and shift (BRIEF-P)	Improvement in working memory and attention (*p* < .001, *d* = 0.23) in the intervention group. Inhibition scores of the BRIEF were stable in the intervention group (*p* = .026, *d* = 0.39). N.S. differences on other BRIEF variables	85%
Lee, 2017 [Canada]	Cogmed intervention for at-term children	Intervention: 12 (n.a) Control: 10 (n.a)	N/A	Intervention: 28.3 (2.3) Control: 38.9 (1.9)	Between 4 and 6 years (mean age: 5.6 years)	Child, Cogmed (JM version), 5 days/week (15 min/session) for 5 weeks	Between 4 and 6 years (mean age: 5.6 years)	Verbal and visuospatial working memory (AWMA) Visual Attention (TOVA) Inhibition, working memory, planning and organizing, emotional control, organization of materials, monitor and shift (BRIEF-P)	N.S. differences between groups on working memory. Improvement in verbal working memory in the intervention group only 5 weeks after training (*p* < .05, *d* = 0.62). N.S. results for visual attention or EF	81%
Anderson, 2018 [Australia]	Placebo intervention for preterm children	Intervention: 45 (51%) Control: 46 (63%)	N/A	Intervention: 27.3 (2.3) Control: 26.9 (1.8)	7 years (mean age: 7.6 years)	Child, Cogmed (RM version), 20–25 sessions (35–50 min/session) over 5–7 weeks	7 years (mean age: 7.6 years)	Attention (TEAch) Inhibition, working memory, planning and organizing, emotional control, organization of materials, monitor and shift (BRIEF-P) Working memory (working memory test battery for children and AWMA)	N.S. on all outcome measures postintervention and up to 24 months posttraining (*d* = N/A)	96%
Grunewaldt, 2013 [Norway]	Waitlist for preterm children; then received Cogmed intervention	Intervention: 9 (66%) Control: 11 (73%)	N/A	Intervention: 29.4 (2.3) Control: 28.2 (3.1)	Between 5 and 6 years (mean age: 5.8 years)	Child, Cogmed (JM version), 5 days/week (10–15 min/session) for 5 weeks	Between 5 and 6 years (mean age: 5.8 years)	Verbal and visual working memory (Digit Span and Spatial Span board task) Attention and hyperactivity (The ADHD Rating Scale-IV) Attention/EF (NEPSY)	Improvement on spatial working memory (*p* = .01, *d* = 0.34). N.S. for verbal working memory. Improvement on attention/EF tasks (*p* = .01, *d* = 0.26). N.S. for ADHD Rating Scale but slight reduction in hyperactivity after training	88%
Grunewaldt, 2016 [Norway]	No intervention for preterm children	Intervention: 20 (70%) Control: 17 (65%)	N/A	Intervention: 28.8 (2.8) Control: 29.6 (2.6)	Between 5 and 6 years (mean age: 5.8 years)	Child, Cogmed (JM version), 5 days/week (10–15 min/session) for 5 weeks	Between 5 and 6 years (mean age: 5.8 years)	Verbal and visual working memory (Digit Span task from WISC and Spatial Span task from WMS) Attention and hyperactivity (The ADHD Rating Scale-IV) Attention/EF (NEPSY)	At 7-month follow-up, significant difference in visual working memory for intervention group (*p* = .003, *d* = 0.23). At 7-month follow-up, n.s. differences between groups for verbal working memory, attention/EF or behavioral measures	77%
Aarnoudse-Moens, 2018 [The Netherlands]	No control group	Intervention: 12 (50%)	N/A	Intervention: 28.7 (1.5)	Between 9 and 11 years (mean age: 10.2 years)	Child, BrainGame Brian, 25 sessions (45 min/session) for 6 weeks	Between 9 and 11 years (mean age: 10.2 years)	Attention (DBD rating scale, Dutch version) Visual working memory (adaptation of a task by Nutley et al.) Inhibition (stop signal task) Cognitive flexibility (stimulus response compatibility task)	Significant improvements in visual working memory after intervention (*p* = .02, d= N/A). N.S. for inhibition, cognitive flexibility and parents rating of attention	66%
van Houdt, 2019 [The Netherlands]	Waitlist for preterm children and placebo intervention for preterm children	Intervention: 29 (55%) Control (waitlist): 30 (33%) Control (placebo): 29 (38%)	N/A	Intervention: 28.2 (1.3) Control (waitlist): 27.8 (1.4) Control (placebo): 28.0 (1.0)	Between 8 and 12 years old (mean age: 10.2 years)	Child, BrainGame Brian, 25 sessions (30–45 min/session) for 6 weeks	Between 8 and 12 years old (mean age: 10.2 years)	Attention (Child-ANT)	N.S. results for attention postintervention (*d* = N/A)	96%
van Houdt, 2021 [The Netherlands]	Waitlist for preterm children and placebo intervention for preterm children	Intervention: 29 (55%) Control (waitlist): 30 (33%) Control (placebo): 29 (38%)	N/A	Intervention: 28.2 (1.3) Control (waitlist): 27.8 (1.4) Control (placebo): 28.0 (1.0)	Between 8 and 12 years old (mean age: 10.2 years)	Child, BrainGame Brian, 25 sessions (30–45 min/session) for 6 weeks	Between 8 and 12 years old (mean age: 10.2 years)	Attention (SWAN questionnaire) Inhibition, working memory, planning and organizing, emotional control, organization of materials, monitor and shift (BRIEF) Verbal working memory (digit span backward subtest of WISC-III) Visuospatial working memory (Grid task backward condition) Inhibition (stop signal task) Cognitive flexibility (MSIT)	N.S. results for attention or EF tasks postintervention (*d* = N/A)	96%
Everts, 2017 [Switzerland]	Waitlist for preterm children	Intervention (memory strategy training): 11 (54%) Intervention (intensive working memory practice): 7 (42%) Control (waitlist): 5 (60%)	N/A	Intervention (memory strategy training): 30 (0.7) Intervention (intensive working memory practice): 29.9 (0.6) Control (waitlist): 28.5 (0.9)	Between 7 and 12 years old (mean age: 10 years)	Child, Memory strategy training (Memo-training), 4 sessions (60 min) over 4 weeks and Child, Intensive working memory practice (Braintwister), 20 sessions (10 min/session), over 4 weeks	Between 7 and 12 years old (mean age: 10 years)	Verbal working memory (WISC letter-number sequencing), visual working memory (MLT spatial positioning), attention (TAP divided attention error), inhibition (TAP Go-no-Go error)	Following memory strategy training, there was significant improvement in verbal (*p* = .02, *d* = N/A) and visual (*p* = .008, *d* = N/A) working memory and inhibition (*p* = .006, *d* = N/A). Following intensive working memory practice, there was significant improvement of verbal working memory (*p* = .015, *d* = N/A) and inhibition (*p* = .027, *d* = N/A)	66%
Siffredi, 2021 [Switzerland]	Waitlist for preterm children then received mindfulness intervention	Intervention: 29 (48.3%) Control: 27 (59.3%)	N/A	Intervention: 29.29 (1.92) Control: 29.12 (1.93)	Between 10 and 14 years old (mean age: 12.1 years)	Child, mindfulness-based intervention, eight sessions (90 min) over 8 weeks	Between 10 and 14 years old (mean age: 12.1 years)	Inhibition, working memory, planning and organizing, emotional control, organization of materials, monitor and shift (BRIEF), Inhibition (Flanker Visual Filtering Task), working memory (Letter-Number sequencing subtest, WISC-IV)	Following the mindfulness-based intervention there was significant improvement in parent-reported executive functioning (*p* ≤ .002, d = N/A), with the high-risk subgroup (lower GA and BW) benefiting more immediately postintervention and the long lasting effect measured more specifically in the moderate-risk group (higher GA and BW). N.s. results for child measured EF	85%

*Note.* AWMA = Automated Working Memory Assessment; BENCI = Battery for Computerized Neuropsychological Evaluation of Children; BRIEF = behavior rating inventory of executive function; BRIEF-P = behavior rating inventory of executive function, preschool version; CBCL = Child Behavior Checklist; Child-ANT = Child version of the Attention Network Test; DBD = Disruptive Behavior Disorders; ECBI = Eyberg Child Behavior Inventory; EF = executive functions; F = Female; MSIT = Multisensory Integration Test; n.s. = nonsignificant; N/A = not applicable/available; NEPSY = a developmental neuropsychological assessment; PEFEN. Programa de Estimulaciòn en Fonciones Ejecutivas para Niños; SWAN = Strengths and Weaknesses of ADHD symptoms and Normal Behavior; TEAch = Test of Everyday Attention for children; TOVA = Test of Variables of Attention; WISC = Wechsler Intelligence Scale for Children 3rd edition; WMS = Wechsler Memory Scale 3rd edition.

A total of 517 premature children (female participants ranging from 23% to 70% of the samples) received an intervention between birth and 14 years old. The duration of intervention ranged from 10 to 150 minutes over the course of 2 weeks to 6 months. The GA of preterm participants ranged from 26 to 35 weeks depending on study criteria. Fourteen studies (82%) included a measure of attention as an outcome. A total of 12 articles (70%) measured at least one subset of executive functions. Specifically, working memory was assessed in nine studies (53%), inhibition in eight articles (47%), planning and organizing in seven articles (41%), and cognitive flexibility was an outcome measure in three studies (17%). A total of five articles (29%) explored the efficacy of an intervention received at infancy (under a year old) on the executive functioning and/or attention skills of children aged 2–9 years old. Contrastingly, most studies (12 studies, 71%) detailed the efficacy of programs implemented at toddlerhood, preschool, school-age, or preadolescence (1.5–14 years old). Table 3 shows the count of studies done for each intervention assessed in this review. Studies detailing the efficacy of Cogmed, a preschool and school-age computerized cognitive training developed by Klingberg and his colleagues (2005) were the most prevalent, representing 23% of all articles (Anderson et al., 2018; Grunewaldt et al., 2013; Grunewaldt et al., 2016; Lee et al., 2017); followed closely by articles reviewing the impact of BrainGame Brian (Prins et al., 2013), another school-age and preadolescence computerized cognitive training program (17%; Aarnoudse-Moens et al., 2018; van Houdt et al., 2019, 2021). Other interventions received during the neonatal and infancy period, such as the Mother–Infant Transaction Program (MITP; Rauh et al., 1990) and the Newborn Individualized Developmental Care and Assessment Program (NIDCAP; Als, 1986) were documented in two articles (11%). Lastly, six programs, including a program received under a year old, the Infant Behavioral Assessment and Intervention Program (IBAIP; Hedlund, 1998), and programs received between 1.5 and 14 years old, such as the parent–child interaction therapy (PCIT; Funderburk and Eyberg, 2011), Therapeutic listening (TL) (Frick and Young, 2019), the Programa de Estimulaciòn en Fonciones Ejecutivas para Niños (PEFEN; Cruz-Quintana et al., 2014), a memory training program (Everts et al., 2017) and a mindfulness-based intervention (Siffredi et al., 2021) were assessed with one study only (about 5% each).

**Table 3.** Overview of different interventions included in studies.

Types of interventions	% (*n*)
Type of the Mother–Infant Transaction Program (MITP; Rauh et al., 1990)	11.76 (2)
The Newborn Individualized Developmental Care and Assessment Program (NIDCAP; Als, 1986)	11.76 (2)
The Infant Behavioral Assessment and Intervention Program (IBAIP; Hedlund, 1998)	5.88 (1)
Parent–child interaction therapy (PCIT; Funderburk and Eyberg, 2011)	5.88 (1)
Therapeutic listening (TL; Frick and Young, 2019)	5.88 (1)
Programa de Estimulaciòn en Fonciones Ejecutivas para Niños (PEFEN, Cruz-Quintana et al., 2014)	5.88 (1)
Cogmed (Klingberg et al., 2005)	23.52(4)
Braingame Brian (Prins et al., 2013)	17.64 (3)
Other type of cognitive training (Everts et al., 2017)	5.88 (1)
Mindfulness-based intervention (Siffredi et al., 2021)	5.88 (1)

The rating of the quality of methodology and outcomes assessing certainty in the body of evidence for all studies ranged from 44% to 96% (reported for each study individually in Tables 1 and 2), with interventions received during infancy and those received in later developmental stages being similarly robust (respective means of 84% and 81% for quality). In terms of risk of bias, 50% of our studies showed an overall high risk of bias, 21.4% showed some concerns and 28.6 were low risk of bias (individual and summarized reports are available in online supplementary Table S2 and online supplementary Figures 1 and 2). The domains showing most bias is the selection of reported results (35.7% high risk and 28.6% showing some concerns) followed by the deviations from intended interventions (28.6% showing some concerns) and the randomization process (7.1% high risk and 14.3% showing some concerns). The bias arising from missing outcome data and measurement of the outcome was more minimal, with only one study showing high risk of bias in each of these domains (7.1%). Three articles could not be assessed using the Cochrane risk of bias tool for randomized trials 2.0 since they did not constitute randomized controlled trials [one included a term-born control group (Lee et al., 2017); one was a follow-up article where more participants were included at a later time to constitute a new control group (Grunewaldt et al., 2016); and one was a pilot study with no control group (Aarnoudse-Moens et al., 2018)].

### Interventions offered during neonatal and infancy period (under 1 year old)

**Figure 3.**
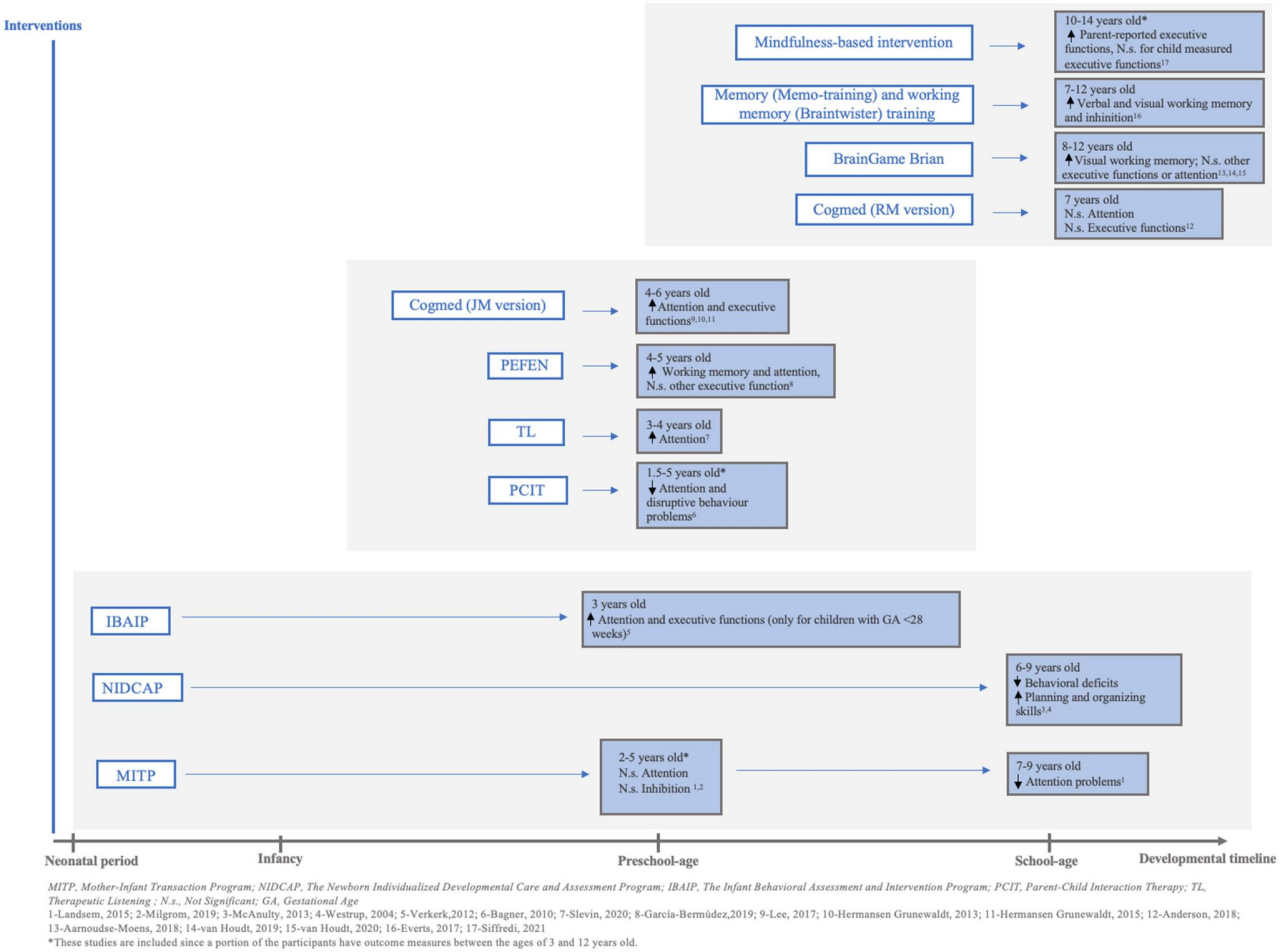
Summary of interventions and results.

Two hundred twenty-eight preterm children whose executive functions and/or attention abilities were measured between 2 and 9 years old received three different types of interventions, both in the NICU and postdischarge. The results were detailed in five different articles. Figure 3 summarizes the results of attention and executive outcomes measured following those interventions.

The MITP developed by Rauh and his colleagues (1990) trains parents to recognize their infant’s physiological states and identify signs of stress, in order to adequately provide sensitive responses that guide their baby back to a homeostatic state. Two articles aimed to evaluate the effect of an MITP-type intervention on the neurodevelopment of children (Landsem et al., 2015; Milgrom et al., 2019). Both interventions were comparable in terms of duration: 60-minute sessions, 10–11 sessions over periods of 13–19 weeks, and a quality rating of 88%. While Milgrom and his colleagues (2019) found no beneficial effects of the intervention on attention and inhibition in children aged 2 and 4.5 years old, Landsem and his co-authors (2015) found less attention problems in the intervention group at 7 and 9 years old, as reported by their mothers, fathers, and teachers, with a moderate effect size (*d* = 0.40–0.62).

The NIDCAP (Als, 1986) aims to adjust the sensory environment and the individualized care of the infants, based on the observation of their tolerance levels and needs (McAnulty et al., 2013; Westrup et al., 2004). One well-rated article (81% quality rating) showed that children who received the NIDCAP intervention during infancy had fewer significant behavioral deficits at 6 years old (Westrup et al., 2004). Similarly, another article (92% quality rating) revealed a positive effect of the NIDCAP intervention on older school-aged children (9 years old), showing overall higher mean scores on a task that assessed planning and organizing skills (McAnulty et al., 2013).

One article examined the effect of the IBAIP (Hedlund, 1998), administered in the neonatal and infancy period, on premature children (70% quality rating; Verkerk et al., 2012). The IBAIP teaches parents to recognize the infant’s strengths and self-regulation skills when interacting with the environment, and to adjust the infant’s surroundings as needed. Overall, no beneficial effect was measured on attention (measured directly with the child or through parent-report questionnaires) or executive functions (objective and subjective measures of inhibition, working memory, planning and organizing) following the IBAIP intervention at 44 months. It is noteworthy that only a subgroup of vulnerable children seems to have benefited from the program. Specifically, significant positive effects were reported by parents on questionnaires that assessed attention and executive functions for children with a GA <28 weeks when compared with premature children receiving standard care (GA mean around 30 weeks; Verkerk et al., 2012).

In sum, among the interventions administered during the neonatal period and infancy, five articles assessed three different programs received by neonates or infants born prematurely. Two of these programs that targeted infants, were detailed in three articles and seemed to positively impact children’s cognitive outcomes, specifically in attention and executive functions. MITP-type interventions and NIDCAP seem to improve attention, behavior, and planning and organizing in children from 6 to 9 years old (Landsem et al., 2015; McAnulty et al., 2013; Westrup et al., 2004). Contrastingly, one study found no significant results when evaluating the efficacy of an MITP-type intervention (Milgrom et al., 2019). Interestingly, one group of researchers found the benefit of the IBAIP program to be limited to a more vulnerable population, the extremely premature children (GA <28 weeks; Verkerk et al., 2012).

### Interventions offered during toddlerhood, preschool and school-age, and preadolescence

Two hundred eighty-nine children took part in eight different intervention programs from the ages of 1.5–14 years old. The results were reported in 12 articles. Figure 3 also includes a summary of the results of interventions administered to toddlers, preschoolers, and school-aged and preadolescent children.

The PCIT teaches parents how to use an authoritative parenting style (i.e., nurturance and good communication, with firm control) with their children in order to reduce disruptive behavior. It is composed of two stages: a child-directed interaction in which the parent praises the child’s appropriate play behavior and parent-directed interaction in which the parent is taught how to respond to the child’s noncompliance (Bagner et al., 2010). One robust study (92% quality rating) demonstrated that parents of children who received the intervention between 18 months and 5 years old found that their child had less attention and disruptive behavior problems at those ages. This clinically significant and large effect (*d* = 1.1–2.3) was, for most of the children, maintained 4 months postintervention (Bagner et al., 2010).

TL is a home music intervention program designed to sharpen selective attention (Slevin et al., 2020). The auditory program is administered 5 times per week for 6 months and consists of listening to modified music on specialized headphones while engaging in regular daily activities. Attention levels were measured pre- and postintervention using the Reynell Attention Scale, which determines the child’s general attention level on a scale of 1 (“fleeting attention”) to 6 (“integrated attention”). Slevin and his colleagues (2020) noted an improvement in attention levels for all children aged between 3 and 4 years old by one level (22% of sample size or two children) and two levels (78% of sample size or seven children) immediately after the intervention. No long-term follow-up outcomes were reported. It is important to bear in mind that this article received the lowest methodological rating (44% quality rating).

PEFEN is an executive function stimulation program, composed of group activities for children that center around working on the components of executive functions (e.g., working memory or inhibition; Cruz-Quintana et al., 2014). It also incorporates mindfulness techniques adapted to children (García-Bermúdez et al., 2019). One article (85% quality rating) evaluating the efficacy of the PEFEN program on children between 4 and 5 years old found that working memory, measured directly in children, improved significantly after the intervention with a small to moderate effect size (*d* = 0.23–0.39). However, results from parental questionnaires did not reflect this improvement, and no significant results were noted when reported by parents (García-Bermúdez et al., 2019).

Four articles examined the effect of Cogmed, a 5- to 7-week-long computerized training program that focuses on working memory. In terms of methodological rigor, the articles ranged from a quality score of 77% to 96%. Three versions of Cogmed are available depending on the age of the child, two of which were assessed in this review. The JM version of Cogmed, designed for preschoolers (4–6 years old) focuses on visuospatial tasks and is composed of short sessions (10–20 min). The RM version of Cogmed, proposed for school-aged children, incorporates longer and more complex exercises (Klingberg et al., 2005). One study found the JM version of Cogmed to yield a positive moderate effect (*d* = 0.62) on verbal working memory in preterm children 5 weeks after the intervention, although these effects were not apparent immediately posttraining. Furthermore, no effect was observed in visuospatial working memory, posttraining or at the 5-week follow-up. Similarly, no change was found in visual attention or other executive functions as measured by parental questionnaires (Lee et al., 2017). Contrastingly, another group of researchers showed the efficacy of the JM version of Cogmed in improving visual working memory of premature children but found no effect on verbal working memory. The program was also beneficial in improving attention, as well as trained and nontrained executive function in these children. Effect sizes were small to moderate ranging from *d* = 0.26 to *d* = 0.34. (Grunewaldt et al., 2013). At the 7-month follow-up, improvement in visual working memory was sustained in preterm children, with a small effect size (*d* = 0.23). However, there were no significant differences in attention or behavioral measures (Grunewaldt et al., 2016). Lastly, Anderson and his colleagues (2018) found no short- or long-term benefit to the RM version of Cogmed on working memory or attention in prematurely born 7-year-old children.

Similar to Cogmed, BrainGame Brian is a computerized executive functioning training program. In addition to training working memory, this program also incorporates impulse control and mental flexibility training. The intervention is designed for school-aged children between the ages of 8 and 12 years old (Prins et al., 2013). One methodologically weak study (66% quality rating), partly because of the lack of a control group, found clinically significant changes in visual working memory after intervention. However, results were not significant for inhibition, cognitive flexibility, nor parents’ rating of attention (Aarnoudse-Moens et al., 2018). Concordant with these results, two other robust articles (96% quality rating) also noted no beneficial effect of BrainGame Brian on attention or executive functions (van Houdt et al., 2019, 2021).

Everts and colleagues (2017) used two different types of programs administered over the course of 4 weeks to very preterm-born school-age and preadolescents aged 7–12 years old: a memory strategy training (Memo-training) and an intensive working memory practice (Braintwister). Following both trainings there was a significant improvement in verbal working memory and inhibition. Visual working memory, however, was only improved in children who received the memory strategy training program. The number of participants ranged from 5 to 11 children, which partially accounted for the low-quality rating score (66%).

Lastly, an 8-week mindfulness-based intervention (weekly 90-min sessions; 85% quality rating) revealed significant improvements that were limited to parent-reported executive functions in very preterm preadolescents and adolescents aged 10–14 years old. However, those positive results did not translate to executive functions as measured directly with the child (Siffredi et al., 2021).

### Summary of results

A count of the studies that showed a positive effect following intervention is presented in Table 4. A total of 517 premature children (GA ranging from 26 to 35 weeks; female participants constituting 23%–70% of the samples; White participants constituting 74%–82% of the samples although only two studies included data on ethnicity) received an intervention between birth and 14 years of age. Eleven different interventions were assessed in 17 studies. Five of the studies focused on interventions administered in the NICU or shortly postdischarge, while 12 other articles reported on interventions received by children between the ages of 1.5 and 14 years old. Of those 17 articles examined, 12 (70%) showed statistically significant results short-term (postintervention). Positive long-term results were reported in three studies (from 3 to 7 months postintervention). Effect sizes were reported in six studies and ranged from small (*d* = 0.23) to large (*d* = 2.3), although most studies showed a small to moderate effect and one study showed a large effect. Positive findings of improved executive functioning were observed in nine studies out of the 12 that included a measure for this cognitive domain (75%). Contrastingly, positive outcomes in attention measures were reported in 6 out of the 14 studies that evaluated this cognitive function (42%). A total of eight articles documented positive results that were based on objective measures (neuropsychological testing) while five studies yielded outcomes based on subjective measures (parents’ and/or teachers’ questionnaires). Similar patterns across included studies contributed to a “high” or “some concerns” risk of bias assessment in the selection of reported results domain mainly: not preregistering the study which led to no prespecified plan regarding measures included and analysis, vague mention of shortened protocols because of lower recruitment target and not including all subscales of a neuropsychological test or questionnaire administered. Several studies omitted the inclusion of lost to follow-up participants from the outcome data (e.g., through the use of an analysis sensitive to missing data, i.e., intention-to-treat analysis), which led to a significant proportion of bias arising from the deviations from intended intervention. Lastly, some studies were lacking important information on their randomization process (allocation sequence concealment details) or used an allocation sequence generation based on availability of participants for a certain intervention date. In addition to these patterns, other characteristics also contributed to lower quality assessment scores. In terms of external validity, most studies were administered and/or assessed by highly trained staff and in extremely specialized facilities, leading to less generalizable results given that these services are not representative of what is accessible to most patients. Moreover, most studies included lacked power given that they did not have a large enough sample size to detect a clinically important effect where the probability value for a difference being due to chance is less than 5%.

**Table 4.** Summary of the number of studies showing a positive effect following intervention.

Types of interventions	Studies showing a positive effect (%)	Studies showing no significant effect
Type of the Mother–Infant Transaction Program (MITP; Rauh et al., 1990)	1 (50%)	1 (50%)
The Newborn Individualized Developmental Care and Assessment Program (NIDCAP; Als, 1986)	2 (100%)	0 (0%)
The Infant Behavioral Assessment and Intervention Program (IBAIP; Hedlund, 1998)	1 (100%)	0 (0%)
Parent–child interaction therapy (PCIT; Funderburk and Eyberg, 2011)	1 (100%)	0 (0%)
Therapeutic listening (TL; Frick and Young, 2019)	1 (100%)	0 (0%)
PEFEN (Cruz-Quintana et al., 2014)	1 (100%)	0 (0%)
Cogmed (Klingberg et al., 2005)	3 (75%)	1 (25%)
Braingame Brian (Prins et al., 2013)	1 (33.4%)	2 (66.6%)
Other type of cognitive training (Everts et al., 2017)	1 (100%)	0 (0%)
Mindfulness-based intervention (Siffredi et al., 2021)	1 (100%)	0 (0%)

## Discussion and conclusion

### Postintervention benefits

Given the plethora of neurodevelopmental consequences of the premature brain (Fleiss et al., 2020), notably on higher-order cognitive functions (i.e., executive functions and attention; Breeman et al., 2016; Taylor and Clark, 2016), the aim of this systematic review was to synthesize the interventions geared toward improving executive functioning and attention skills measured in premature children during their preschool and school years (3–12 years old). We systematically reviewed 17 studies that assessed 11 intervention programs. Overall, all of the 11 interventions administered yielded positive outcomes in at least one study, which supports the benefit of such programs. Twelve articles (70%) showed positive outcomes on cognition in the short-term (postintervention), while three showed consolidated effects in the long-term (3–7 months postintervention). Although the impacts of the interventions were reported for executive functions as well as attention, more programs were reported as being beneficial in improving executive functions specifically (75% for executive function and 42% for attention). In fact, most improvements were observed in working memory, with 77% of studies that measured working memory showing an improvement after intervention. This result is not surprising given that the two interventions documented in almost half of the studies (Cogmed and BrainGame Brian) were working memory training. Two out of the three studies that focused on cognitive flexibility also reported improvements following the intervention. Lastly, some benefits were observed in planning and organizing skills, as well as inhibition. However, the efficacy rates per study are lower than for other executive function subsets (42% and 37%, respectively). Improvements in attention and executive functions were measured directly with child assessments, as well as reflected in parent’s and teacher’s perception of the child. Effect sizes ranged from small to moderate, with only one study showing large effects (PCIT; Bagner et al., 2010). However, the studies reported had modest sample sizes (9–76 participants), which does not allow for the detection of a true effect size.

### Comparison of the programs

The MITP, administered in the NICU and documented in two distinct, methodologically robust studies (88% quality rating for both) that had comparable interventions in terms of age of administration, structure, and duration, revealed surprisingly discrepant results (Landsem et al., 2015; Milgrom et al., 2019). Overall, the program revealed improvements in the attention skills of older children (school-aged, 7 and 9 years old), as perceived by parents and teachers (Landsem et al., 2015). However, these results were not immediately apparent in preschool-aged children (Landsem et al., 2015; Milgrom et al., 2019). In contrast, the NIDCAP (Als, 1986), another program administered in the NICU, showed conclusive positive results in two studies (81% and 92% quality rating), improving both executive functions and attention in school-aged children (6–9 years old; McAnulty et al., 2013; Westrup et al., 2004). Lastly, the IBAIP (Hedlund, 1998), also administered in the NICU, showed improvements in executive functions limited to an especially vulnerable group of very prematurely born preschoolers (70% quality rating; Verkerk et al., 2012). In summary, of the three NICU interventions examined, the NIDCAP seemed to yield the most conclusive results, which spanned over several cognitive domains (executive functions and attention). On the other hand, the MITP showed results limited to attention skills in school-aged children, and the IBAIP, the lowest rated article in terms of quality of methodology, seemed to benefit executive function in a subgroup of very premature preschoolers.

Three different interventions were administered to preschool-aged children between 1.5 and 5 years old and were each reported in a single study. The PCIT (Bagner et al., 2010) and TL (Slevin et al., 2020) both yielded improvements in attention and disruptive behaviors. However, the effect of PCIT was maintained 4 months postprogram, had a statistically large effect size, and its protocol had a higher methodological quality score (92% quality rating as opposed to 44% for TL), rendering it more replicable for future studies. Lastly, PEFEN (García-Bermúdez et al., 2019, 85% quality score) showed positive small to moderate results and improved executive functions in preschoolers. In short, although the methodological quality varied across studies, all interventions administered to preschoolers seemed beneficial in improving attention and executive functioning.

On the other hand, Cogmed was examined in four different studies, ranging from 77% to 96% on their methodological scores, administered to children aged 4–7 years old. Overall, Cogmed showed mixed results in improving executive functioning and attention in premature school-aged children. While some studies demonstrated improvements in working memory following the training of said function, only one study exhibited a broader effect on other executive functions and cognitive skills, and these results were not sustained in the long-term (Anderson et al., 2018; Grunewaldt et al., 2013, 2016; Lee et al., 2017). BrainGame Brian, another computerized executive functioning training program, is administered to older children aged between 8 and 12 years old. However, the results were once again limited to an improvement in working memory, although the training is more exhaustive as compared to Cogmed as it also includes training in impulse control and mental flexibility. The positive effect on working memory was also apparent in a single, lower-rated study in terms of quality (66%, instead of 96% for the other two) limiting the reproducibility of these results (Aarnoudse-Moens et al., 2018; van Houdt et al., 2019, 2021). To conclude, computerized cognitive training seems to provide little evidence of improving executive functions and attention in school-aged and preadolescent preterm children. The positive results of training apply mostly to working memory and are limited in terms of the potential to generalize to other cognitive functions. This is concordant with literature on the use of computerized cognitive training in other clinical populations (i.e., ADHD symptoms) that show small to moderate improvements in working memory that did not transfer to other neuropsychological domains (Westwood et al., 2023). This effect was also observed in neurotypical populations where narrow transfers appear following computerized training (i.e., some studies find slight improvements in attention; Diamond and Ling, 2016).

Alternatively, the two different types of working memory and memory training (memo-training and braintwister) presented by Everts and colleagues (2017) showed promising results in their sample group. However more data is required, especially given their small sample size (between 5 and 11 per group) and the methodological quality score of 66%. Lastly, while parents documented positive changes in their preadolescent’s executive functions following a mindfulness-based intervention, this program did not yield significant results in terms of objective and direct measures with the child (85% quality score; Siffredi et al., 2021).

Overall, several different types of interventions were reviewed: child-parent interactions (NICU- and non-NICU-based), music therapy, computerized training programs, and mindfulness and memory techniques. These programs diverge on several dimensions: parental engagement, focus on very early development, personalization of intervention, feedback timing and specificity and ecological reproducibility factors. The theoretical frameworks underlying these interventions range from research on child development (attachment theory in child-parent interactions) to neurobiology (physiological effect of stress and relation to cognition). Thus, the heterogeneous underlying mechanisms targeted to improve executive and attentional functioning (i.e., cognitive development, emotional regulation, and social skills) could account for the observed differences in the efficacy of programs.

### Overall noteworthy characteristics of all tested interventions (e.g., duration, construct, resources)

Given the wide range of interventions reviewed, it is important to note the different structures and characteristics of the programs. In terms of duration, the shortest intervention (Braintwister; Everts et al., 2017) that yielded positive effects lasted 4 weeks and involved a weekly hour-long session. Some other successful programs had a duration of 6 months (TL; Slevin et al., 2020). Thus, the present findings only permit limited interpretations of the duration of an effective intervention, given that we are unable to determine if longer programs would result in more improvements. In fact, the summary of the literature is presented using a categorical approach (i.e., is the intervention effective or not) rather than a dimensional classification that would clarify not only if the intervention is effective, but how effective it is, particularly when compared to each other which would require a meta-analysis. Addressing the question of duration and intensity of the intervention is crucial for the preterm population given that studies have indicated that lengthier interventions tend to produce more favorable outcomes in neurotypical population (Diamond and Ling, 2016). Furthermore, most programs require a vast number of resources to implement. For example, some interventions are carried out by a team of multidisciplinary professionals in specialized hospital settings, while others call for great parental involvement. Yet, these resources could be sparser in rural areas or in communities with lower socioeconomic status. Consequently, it is important to consider socioeconomic factors when evaluating the efficacy of such interventions.

### Interaction between age and efficacy of programs

Based on the efficacy of the programs detailed above, interventions administered at preschool age seemed to yield the most conclusive results in improving executive functions and attention in preterm children, although these results should be interpreted with caution given the descriptive nature of this review and the heterogeneity of the interventions compared. Nonetheless, these results are concordant with the literature, which highlights an exponential development of these high-level functions at the preschool age, offering a favorable interventional window and increasing the impact of administering such programs at this age (Diamond, 2020). Although the interventions at the preschool age showed the most promising results, the programs were the most heterogeneous compared to other developmental periods. Interventions were related to child-parent interactions, music therapy, an executive function stimulation program with components of mindfulness and a computerized executive function program. This potentially reinforces the idea of the preschool age as the most ideal period to implement changes given that several underlying mechanisms targeted were effective in improving executive functions and attention at this age. In contrast, training received at a very young age, in the NICU, or later, at school age, demonstrated mixed results in the premature population. This could suggest that interventions in the NICU are provided too early into brain development, given that the structures that support the development of attention and executive functions are still too immature (Diamond, 2020). Programs targeting older children (school-aged) might be overdue, considering that some cognitive functions might be solidified by then (Diamond, 2020).

Importantly, one study examining a NICU program found an interaction between gestational age and the efficacy of the program, noting the positive effect of the intervention only on a subgroup of very vulnerable preterm children (GA < 28 weeks; Verkerk et al., 2012). Conversely, two NICU studies, similar in terms of methodology, revealed different results for a population of very preterm (GA mean <28 weeks) and moderately preterm children (GA mean >30 weeks; Landsem et al., 2015; Milgrom et al., 2019), showing improvements only on the moderately preterm children. This highlights the important interaction between the impact of the intervention and the GA, particularly for NICU programs, although the direction of the relationship remains unclear. This interaction is not apparent in preterm preschool- or school-aged children, suggesting that at these developmental stages, all preterm children might benefit from attention and executive intervention strategies, regardless of the level of prematurity.

### Methodological limitations and conclusion

This review focused on summarizing the results of NICU-based interventions as well as programs administered to preschool or school-aged children born prematurely. When examining programs where the intervention was NICU-based and the outcome measure was taken at a later developmental stage, it is important to note the difficulty of controlling for the effect of confounding factors (i.e., GA, sex, socioeconomic status, other school and family environmental factors such as parenting style, etc.) on the efficacy of the interventions.

Moreover, due to the heterogeneity of the various programs reviewed, ranging from interventions in the NICU to child-parent interactions and computerized training, as well as the diverseness in the age at administration of the programs, measurements of outcomes (i.e. objective and direct cognitive tests or parental-reported questionnaires), and the observed outcomes on cognition or generalized daily behaviors, the findings presented should be interpreted as descriptive results. Although this review provides a thorough scan of the literature, quantitative analyses of the results (i.e., statistical analyses in a meta-analysis) should be performed in a future study, when the literature will include more comparable studies in terms of intervention features, outcome measures and age at intervention.

Furthermore, for the advancement of the literature and in regard to the questions raised at the outset, there is a pressing need for large-scale and rigorously controlled studies that allow for sufficient power to detect true effect sizes. Additionally, this review considered studies published in English or French, with the results showing that all studies were published in 10 Western nations. Future reviews should aim to encompass studies in multiple languages across a diverse range of countries, including non-Western regions, to ensure a more inclusive representation of global perspectives in this field of research and mitigate potential language-related biases. Authors should also systematically report important participant characteristics (i.e., ethnicity and gender), as well as include these variables in their analysis, to ensure generalizability and applicability across diverse populations. To minimize the risk of bias, studies should be preregistered with a specific plan for measures to be included and analyses to be conducted. To ensure a low risk of bias in terms of the selection of reported results, all subscales of a measure should be included in the results when published. Sensitive analyses should also be included when missing data is reported.

To our knowledge, this is the first systematic review to summarize the interventions aimed at improving executive functioning and attention in preschool and school-aged children born prematurely. Moreover, this review emphasizes the importance of focusing primarily on preschool and possibly on school-aged populations, given the critical effects of early and timely intervention. Although some of the programs show clear improvements in executive and attention functioning, an individualized selection of the various interventions available should be performed according to the target population before implementing these program protocols in clinical settings. Future research should focus on reviewing interventions aiming at improving executive and attentional functioning during the adolescence and young adulthood period. This would enable comparison with our findings and offer valuable insights into the effectiveness of such interventions during different developmental stages. Alternative avenues of research could also delve into the underlying mechanisms driving the efficacy of these interventions. This may involve investigating cognitive, social, or emotional development in relation to developing brain structures and functions following such interventions.

## Supplementary Material

jsae068_Supplementary_Data

## Appendix

**Appendix A.** Full search strategy of each database.

Database	Search Strategy
PubMed (NLM)	1. Child[mh] OR Pediatrics[Mh] OR preschool*[tiab] OR pre school*[tiab] OR child*[tiab] OR kid[tiab] OR kid’[tiab] OR kids[tiab] OR kid’s[tiab] OR boy[tiab] OR boy’[tiab] OR boys[tiab] OR boy’s[tiab] OR girl*[tiab] OR schoolchild*[tiab] OR schoolage*[tiab] OR preadolescen*[tiab] OR pre adolescen*[tiab] OR preteen*[tiab] OR pre teen*[tiab] OR tween*[tiab] OR prepubescen*[tiab] OR pre pubescen*[tiab] OR paediatric*[tiab] OR pediatric*[tiab] OR preschool*[OT] OR pre school*[OT] OR child*[OT] OR kid[OT] OR kid’[OT] OR kids[OT] OR kid’s[OT] OR boy[OT] OR boy’[OT] OR boys[OT] OR boy’s[OT] OR girl*[OT] OR schoolchild*[OT] OR schoolage*[OT] OR preadolescen*[OT] OR pre adolescen*[OT] OR preteen*[OT] OR pre teen*[OT] OR tween*[OT] OR prepubescen*[OT] OR pre pubescen*[OT] OR paediatric*[OT] OR pediatric*[OT] 2. “Exercise”[Mh] OR “Exercise Therapy”[Mh] OR “Exercise Movement Techniques”[Mh] OR “Early Intervention, Educational”[Mh] OR “rehabilitation”[Mh: noexp] OR “rehabilitation”[sh] OR “Cognitive Behavioral Therapy”[Mh] OR “Cognitive Remediation”[Mh] OR “Parent-Child Relations”[Mh] OR “Physical activit*”[tiab] OR “Physical Conditioning”[tiab] OR “Physical Education”[tiab] OR “Exercise*”[tiab] OR “Movement Techni*”[tiab] OR “Movement Therap*”[tiab] OR “Motion Techni*”[tiab] OR “Motion Therap*”[tiab] OR “kinesiotherap*”[tiab] OR “kinesitherap*”[tiab] OR “Training”[tiab] OR “Cognitive abilit*”[tiab] OR “Intervention*”[tiab] OR “Program”[tiab] OR “Programs”[tiab] OR “Rehabilitation*”[tiab] OR “Readaptation*”[tiab] OR “Psychosocial”[tiab] OR “Psycho social”[tiab] OR “cognitive stimulation*”[tiab] OR “mindful*”[tiab] OR “mind-ful*”[tiab] OR “behavioral therap*”[tiab] OR “behavioural therap*”[tiab] OR “behavior therap*”[tiab] OR “behaviour therap*”[tiab] OR “cognition therap*”[tiab] OR “cognitive therap*”[tiab] OR “behavioral psychotherap*”[tiab] OR “behavioural psychotherap*”[tiab] OR “behavioral psycho-therap*”[tiab] OR “behavioural psycho-therap*”[tiab] OR “cognition psychotherap*”[tiab] OR “cognitive psychotherap*”[tiab] OR “cognition psycho-therap*”[tiab] OR “cognitive psycho-therap*”[tiab] OR “CBT”[tiab] OR “neurodevelopmental therap*”[tiab] OR “neuropsychological therap*”[tiab] OR “neurocognitive therap*”[tiab] OR “neurodevelopmental psychotherap*”[tiab] OR “neurocognitive psychotherap*”[tiab] OR “cognitive remediation”[tiab] OR “sport*”[tiab] OR “fitness”[tiab] OR “psychoeducation*”[tiab] OR “psycho-education*”[tiab] OR “occupational therap*”[tiab] OR “parent child”[tiab] OR “mother child”[tiab] OR “father child”[tiab] OR “Physical activit*”[OT] OR “Physical Conditioning”[OT] OR “Physical Education”[OT] OR “Exercise*”[OT] OR “Movement Techni*”[OT] OR “Movement Therap*”[OT] OR “Motion Techni*”[OT] OR “Motion Therap*”[OT] OR “kinesiotherap*”[OT] OR “kinesitherap*”[tiab] OR “Training”[OT] OR “Cognitive abilit*”[OT] OR “Intervention*”[OT] OR “Program”[OT] OR “Programs”[OT] OR “Rehabilitation*”[OT] OR “Readaptation*”[OT] OR “Psychosocial”[OT] OR “Psycho social”[OT] OR “cognitive stimulation*”[OT] OR “mindful*”[OT] OR “mind-ful*”[OT] OR “behavioral therap*”[OT] OR “behavioural therap*”[OT] OR “behavior therap*”[OT] OR “behaviour therap*”[OT] OR “cognition therap*”[OT] OR “cognitive therap*”[OT] OR “behavioral psychotherap*”[OT] OR “behavioural psychotherap*”[OT] OR “behavioral psycho-therap*”[OT] OR “behavioural psycho-therap*”[OT] OR “cognition psychotherap*”[OT] OR “cognitive psychotherap*”[OT] OR “cognition psycho-therap*”[OT] OR “cognitive psycho-therap*”[OT] OR “CBT”[OT] OR “neurodevelopmental therap*”[OT] OR “neuropsychological therap*”[OT] OR “neurocognitive therap*”[OT] OR “neurodevelopmental psychotherap*”[OT] OR “neurocognitive psychotherap*”[OT] OR “cognitive remediation”[OT] OR “sport*”[OT] OR “fitness”[OT] OR “psychoeducation*”[OT] OR “psycho-education*”[OT] OR “occupational therap*”[OT] OR “parent child”[OT] OR “mother child”[OT] OR “father child”[OT] 3. Executive Function[Mh] OR Problem solving[Mh: noexp] OR Attention[Mh] OR Memory[Mh] OR Inhibition, Psychological[Mh] OR Metacognition[Mh] OR Impulsive Behavior[Mh] OR Executive Function* [tiab] OR Executive Control*[tiab] OR Problem solving[tiab] OR Reasoning[tiab] OR Attention*[tiab] OR planning[tiab] OR planification[tiab] OR Focus[tiab] OR Concentrat*[tiab] OR Memor*[tiab] OR Recognition[tiab] OR Recall[tiab] OR Inhibition*[tiab] OR Inhibitory[tiab] OR Organiz*[tiab] OR Organis*[tiab] OR cognitive flexibilit*[tiab] OR task switching[tiab] OR cognitive shifting[tiab] OR set-shifting[tiab] OR cognitive control[tiab] OR metacogniti*[tiab] OR meta-cogniti*[tiab] OR Metaemotion*[tiab] OR Meta-emotion*[tiab] OR Metamemor*[tiab] OR Meta-memor*[tiab] OR impulsiv*[tiab] OR Executive Function* [OT] OR Executive Control*[OT] OR Problem solving[OT] OR Reasoning[OT] OR Attention*[OT] OR planning[OT] OR planification[OT] OR Focus[OT] OR Concentrat*[OT] OR Memor*[OT] OR Recognition[OT] OR Recall[OT] OR Inhibition*[OT] OR Inhibitory[OT] OR Organiz*[OT] OR Organis*[OT] OR cognitive flexibilit*[OT] OR task switching[OT] OR cognitive shifting[OT] OR set-shifting[OT] OR cognitive control[OT] OR metacogniti*[OT] OR meta-cogniti*[OT] OR Metaemotion*[OT] OR Meta-emotion*[OT] OR Metamemor*[OT] OR Meta-memor*[OT] OR impulsiv*[OT] 4. Infant, Premature[Mh] OR Infant, Low Birth Weight[Mh] OR Premature Birth[Mh] OR Fetal Growth Retardation[Mh] OR Prematur*[tiab] OR Pre matur*[tiab] OR Preemie*[tiab] OR Premmie*[tiab] OR Preterm[tiab] OR Pre term[tiab] OR Low birth weight*[tiab] OR Low birthweight*[tiab] OR Lowbirth weight*[tiab] OR LBW[tiab] OR VLBW[tiab] OR ELBW[tiab] OR Fetal Growth Retardation[tiab] OR Fetal Growth Restriction[tiab] OR Intrauterine Growth Retardation[tiab] OR Intrauterine Growth Restriction[tiab] OR Intra-uterine Growth Retardation[tiab] OR Intra-uterine Growth Restriction[tiab] OR “Small for Gestational Age”[tiab] OR “Small for Age”[tiab] OR “Small for date*”[tiab] OR Prematur*[OT] OR Pre matur*[OT] OR Preemie*[OT] OR Premmie*[OT] OR Preterm[OT] OR Pre term[OT] OR Low birth weight*[OT] OR Low birthweight*[OT] OR Lowbirth weight*[OT] OR LBW[OT] OR VLBW[OT] OR ELBW[OT] OR Fetal Growth Retardation[OT] OR Fetal Growth Restriction[OT] OR Intrauterine Growth Retardation[OT] OR Intrauterine Growth Restriction[OT] OR Intra-uterine Growth Retardation[OT] OR Intra-uterine Growth Restriction[OT] OR “Small for Gestational Age”[OT] OR “Small for Age”[OT] OR “Small for date*”[OT] 5. Drug Therapy[Mh] OR Drug Therapy[sh] OR Drug*[tiab] OR Medication*[tiab] OR Pharmaco*[tiab] OR Drug*[OT] OR Medication*[OT] OR Pharmaco*[OT] Combination and Limitation: (#1 AND #2 AND #3 AND #4 AND (English [La] OR French[La])) NOT #5
Ovid Medline	1. Exp Child/OR Pediatrics/OR (preschool* OR pre school* OR child* OR kid OR kid’ OR kids OR kid’s OR boy OR boy’ OR boys OR boy’s OR girl* OR schoolchild* OR schoolage* OR preadolescen* OR pre adolescen* OR preteen* OR pre teen* OR tween* OR prepubescen* OR pre pubescen* OR paediatric* OR pediatric*).ti, ab, kw, kf 2. Exp Exercise/OR Exp Exercise Therapy/OR Exp Exercise Movement Techniques/OR Exp Early Intervention, Educational/OR Rehabilitation/OR Rehabilitation.fs OR Exp Cognitive Behavioral Therapy/OR Exp Cognitive Remediation/OR Exp Parent-Child Relations/OR (Physical activit* OR Physical Conditioning OR Physical Education OR Exercise* OR Movement Techni* OR Movement Therap* OR Motion Techni* OR Motion Therap* OR kinesiotherap* OR kinesitherap* OR Training OR Cognitive abilit* OR Intervention* OR Program OR Programs OR Rehabilitation* OR Readaptation* OR Psychosocial OR Psycho social OR cognitive stimulation* OR mindful* OR mind-ful* OR behavioral therap* OR behavioural therap* OR behavior therap* OR behaviour therap* OR cognition therap* OR cognitive therap* OR behavioral psychotherap* OR behavioural psychotherap* OR behavioral psycho-therap* OR behavioural psycho-therap* OR cognition psychotherap* OR cognitive psychotherap* OR cognition psycho-therap* OR cognitive psycho-therap* OR CBT OR neurodevelopmental therap* OR neuropsychological therap* OR neurocognitive therap* OR neurodevelopmental psychotherap* OR neurocognitive psychotherap* OR cognitive remediation OR sport* OR fitness OR psychoeducation* OR psycho-education* OR occupational therap* OR parent child OR mother child OR father child).ti, ab, kw, kf 3. Exp Executive Function/OR Problem solving/OR Exp Attention/OR Exp Memory/OR Exp Inhibition, Psychological/OR Exp Metacognition/OR Exp Impulsive Behavior/OR (Executive Function* OR Executive Control* OR Problem solving OR Reasoning OR Attention* OR planning OR planification OR Focus OR Concentrat* OR Memor* OR Recognition OR Recall OR Inhibition* OR Inhibitory OR Organiz* OR Organis* OR cognitive flexibilit* OR task switching OR cognitive shifting OR set-shifting OR cognitive control OR metacogniti* OR meta-cogniti* OR Metaemotion* OR Meta-emotion* OR Metamemor* OR Meta-memor* OR impulsiv*).ti, ab, kw, kf 4. Exp Infant, Premature/OR Exp Infant, Low Birth Weight/OR Exp Premature Birth/OR Exp Fetal Growth Retardation/OR (Prematur* OR Pre matur* OR Preemie* OR Premmie* OR Preterm OR Pre term OR Low birth weight* OR Low birthweight* OR Lowbirth weight* OR LBW OR VLBW OR ELBW OR Fetal Growth Retardation OR Fetal Growth Restriction OR Intrauterine Growth Retardation OR Intrauterine Growth Restriction OR Intra-uterine Growth Retardation OR Intra-uterine Growth Restriction OR Small for Gestational Age OR Small for Age OR Small for date*).ti, ab, kw, kf 5. Exp Drug Therapy/OR Drug Therapy.fs OR (Drug* OR Medication* OR Pharmaco*).ti, ab, kw, kf Combination and Limitation: (1 AND 2 AND 3 AND 4 AND (English OR French).lg) NOT 5
Ovid All EBM Reviews	1. Exp Child/OR Pediatrics/OR (preschool* OR pre school* OR child* OR kid OR kid’ OR kids OR kid’s OR boy OR boy’ OR boys OR boy’s OR girl* OR schoolchild* OR schoolage* OR preadolescen* OR pre adolescen* OR preteen* OR pre teen* OR tween* OR prepubescen* OR pre pubescen* OR paediatric* OR pediatric*).ti, ab, kw, kf 2. Exp Exercise/OR Exp Exercise Therapy/OR Exp Exercise Movement Techniques/OR Exp Early Intervention, Educational/OR Rehabilitation/OR Rehabilitation.fs OR Exp Cognitive Behavioral Therapy/OR Exp Cognitive Remediation/OR Exp Parent-Child Relations/OR (Physical activit* OR Physical Conditioning OR Physical Education OR Exercise* OR Movement Techni* OR Movement Therap* OR kinesiotherap* OR kinesitherap* OR Motion Techni* OR Motion Therap* OR Training OR Cognitive abilit* OR Intervention* OR Program OR Programs OR Rehabilitation* OR Readaptation* OR Psychosocial OR Psycho social OR cognitive stimulation* OR mindful* OR mind-ful* OR behavioral therap* OR behavioural therap* OR behavior therap* OR behaviour therap* OR cognition therap* OR cognitive therap* OR behavioral psychotherap* OR behavioural psychotherap* OR behavioral psycho-therap* OR behavioural psycho-therap* OR cognition psychotherap* OR cognitive psychotherap* OR cognition psycho-therap* OR cognitive psycho-therap* OR CBT OR neurodevelopmental therap* OR neuropsychological therap* OR neurocognitive therap* OR neurodevelopmental psychotherap* OR neurocognitive psychotherap* OR cognitive remediation OR sport* OR fitness OR psychoeducation* OR psycho-education* OR occupational therap* OR parent child OR mother child OR father child).ti, ab, kw, kf 3. Exp Executive Function/OR Problem solving/OR Exp Attention/OR Exp Memory/OR Exp Inhibition, Psychological/OR Exp Metacognition/OR Exp Impulsive Behavior/OR (Executive Function* OR Executive Control* OR Problem solving OR Reasoning OR Attention* OR planning OR planification OR Focus OR Concentrat* OR Memor* OR Recognition OR Recall OR Inhibition* OR Inhibitory OR Organiz* OR Organis* OR cognitive flexibilit* OR task switching OR cognitive shifting OR set-shifting OR cognitive control OR metacogniti* OR meta-cogniti* OR Metaemotion* OR Meta-emotion* OR Metamemor* OR Meta-memor* OR impulsiv*).ti, ab, kw, kf 4. Exp Infant, Premature/OR Exp Infant, Low Birth Weight/OR Exp Premature Birth/OR Exp Fetal Growth Retardation/OR (Prematur* OR Pre matur* OR Preemie* OR Premmie* OR Preterm OR Pre term OR Low birth weight* OR Low birthweight* OR Lowbirth weight* OR LBW OR VLBW OR ELBW OR Fetal Growth Retardation OR Fetal Growth Restriction OR Intrauterine Growth Retardation OR Intrauterine Growth Restriction OR Intra-uterine Growth Retardation OR Intra-uterine Growth Restriction OR Small for Gestational Age OR Small for Age OR Small for date*).ti, ab, kw, kf 5. Exp Drug Therapy/OR Drug Therapy.fs OR (Drug* OR Medication* OR Pharmaco*).ti, ab, kw, kf Combination and Limitation: (1 AND 2 AND 3 AND 4 AND (English OR French).lg) NOT 5
Ovid Embase	1. Child/OR preschool child/OR school child/OR Pediatrics/OR (preschool* OR pre school* OR child* OR kid OR kid’ OR kids OR kid’s OR boy OR boy’ OR boys OR boy’s OR girl* OR schoolchild* OR schoolage* OR preadolescen* OR pre adolescen* OR preteen* OR pre teen* OR tween* OR prepubescen* OR pre pubescen* OR paediatric* OR pediatric*).ti, ab, kw 2. Exp Exercise/OR Exp kinesiotherapy/OR early childhood intervention/OR Rehabilitation/OR rh.fs OR Exp Cognitive Behavioral Therapy/OR Exp Cognitive Remediation therapy/OR Exp child parent relation/OR Occupational Therapy/OR exp Mindfulness/OR (Physical activit* OR Physical Conditioning OR Physical Education OR Exercise* OR Movement Techni* OR Movement Therap* OR Motion Techni* OR Motion Therap* OR kinesiotherap* OR kinesitherap* OR Training OR Cognitive abilit* OR Intervention* OR Program OR Programs OR Rehabilitation* OR Readaptation* OR Psychosocial OR Psycho social OR cognitive stimulation* OR mindful* OR mind-ful* OR behavioral therap* OR behavioural therap* OR behavior therap* OR behaviour therap* OR cognition therap* OR cognitive therap* OR behavioral psychotherap* OR behavioural psychotherap* OR behavioral psycho-therap* OR behavioural psycho-therap* OR cognition psychotherap* OR cognitive psychotherap* OR cognition psycho-therap* OR cognitive psycho-therap* OR CBT OR neurodevelopmental therap* OR neuropsychological therap* OR neurocognitive therap* OR neurodevelopmental psychotherap* OR neurocognitive psychotherap* OR cognitive remediation OR sport* OR fitness OR psychoeducation* OR psycho-education* OR occupational therap* OR parent child OR mother child OR father child).ti, ab, kw 3. Executive Function/OR Problem solving/OR Exp Attention/OR Exp Memory/OR Exp “inhibition (psychology)”/OR Exp Metacognition/OR impulsiveness/OR (Executive Function* OR Executive Control* OR Problem solving OR Reasoning OR Attention* OR planning OR planification OR Focus OR Concentrat* OR Memor* OR Recognition OR Recall OR Inhibition* OR Inhibitory OR Organiz* OR Organis* OR cognitive flexibilit* OR task switching OR cognitive shifting OR set-shifting OR cognitive control OR metacogniti* OR meta-cogniti* OR Metaemotion* OR Meta-emotion* OR Metamemor* OR Meta-memor* OR impulsiv*).ti, ab, kw 4. prematurity/OR Exp low birth weight/OR Exp intrauterine growth retardation/OR (Prematur* OR Pre matur* OR Preemie* OR Premmie* OR Preterm OR Pre term OR Low birth weight* OR Low birthweight* OR Lowbirth weight* OR LBW OR VLBW OR ELBW OR Fetal Growth Retardation OR Fetal Growth Restriction OR Intrauterine Growth Retardation OR Intrauterine Growth Restriction OR Intra-uterine Growth Retardation OR Intra-uterine Growth Restriction OR Small for Gestational Age OR Small for Age OR Small for date*).ti, ab, kw 5. Exp Drug Therapy/OR dt.fs OR (Drug* OR Medication* OR Pharmaco*).ti, ab, kw Combination and Limitation: (1 AND 2 AND 3 AND 4 AND (English OR French).lg) NOT 5
Ovid PsycINFO	1. 160.ag OR 180.ag OR Pediatrics/OR (preschool* OR pre school* OR child* OR kid OR kid’ OR kids OR kid’s OR boy OR boy’ OR boys OR boy’s OR girl* OR schoolchild* OR schoolage* OR preadolescen* OR pre adolescen* OR preteen* OR pre teen* OR tween* OR prepubescen* OR pre pubescen* OR paediatric* OR pediatric*).ti, ab, id 2. Exp Exercise/OR Movement Therapy/OR Early Intervention/OR Rehabilitation/OR Exp Cognitive Behavior Therapy/OR Cognitive Rehabilitation/OR Exp Parent-Child Relations/OR Occupational Therapy/OR Mindfulness/OR (Physical activit* OR Physical Conditioning OR Physical Education OR Exercise* OR Movement Techni* OR Movement Therap* OR kinesiotherap* OR kinesitherap* OR Motion Techni* OR Motion Therap* OR Training OR Cognitive abilit* OR Intervention* OR Program OR Programs OR Rehabilitation* OR Readaptation* OR Psychosocial OR Psycho social OR cognitive stimulation* OR mindful* OR mind-ful* OR behavioral therap* OR behavioural therap* OR behavior therap* OR behaviour therap* OR cognition therap* OR cognitive therap* OR behavioral psychotherap* OR behavioural psychotherap* OR behavioral psycho-therap* OR behavioural psycho-therap* OR cognition psychotherap* OR cognitive psychotherap* OR cognition psycho-therap* OR cognitive psycho-therap* OR CBT OR neurodevelopmental therap* OR neuropsychological therap* OR neurocognitive therap* OR neurodevelopmental psychotherap* OR neurocognitive psychotherap* OR cognitive remediation OR sport* OR fitness OR psychoeducation* OR psycho-education* OR occupational therap* OR parent child OR mother child OR father child).ti, ab, id 3. Exp Executive Function/OR Problem solving/OR Exp Attention/OR Exp Memory/OR Behavioral Inhibition/OR “Inhibition (Personality)”/OR Metacognition/OR Impulsiveness/OR (Executive Function* OR Executive Control* OR Problem solving OR Reasoning OR Attention* OR planning OR planification OR Focus OR Concentrat* OR Memor* OR Recognition OR Recall OR Inhibition* OR Inhibitory OR Organiz* OR Organis* OR cognitive flexibilit* OR task switching OR cognitive shifting OR set-shifting OR cognitive control OR metacogniti* OR meta-cogniti* OR Metaemotion* OR Meta-emotion* OR Metamemor* OR Meta-memor* OR impulsiv*).ti, ab, id 4. Premature Birth/OR (Prematur* OR Pre matur* OR Preemie* OR Premmie* OR Preterm OR Pre term OR Low birth weight* OR Low birthweight* OR Lowbirth weight* OR LBW OR VLBW OR ELBW OR Fetal Growth Retardation OR Fetal Growth Restriction OR Intrauterine Growth Retardation OR Intrauterine Growth Restriction OR Intra-uterine Growth Retardation OR Intra-uterine Growth Restriction OR Small for Gestational Age OR Small for Age OR Small for date*).ti, ab, id 5. Exp Drug Therapy/OR (Drug* OR Medication* OR Pharmaco*).ti, ab, id Combination and Limitation: (1 AND 2 AND 3 AND 4 AND (English OR French).lg) NOT 5

**Appendix B.** Modified Downs and Black checklist for the assessment of methodological quality of both randomized and nonrandomized studies (Downs and Black, 1998).

Item	Criteria	Possible answers
1	Reporting Is the hypothesis/aim/objective of the study clearly described?	Yes = 1 No = 0
2	Are the main outcomes to be measured clearly described in the Introduction or Methods section? If the main outcomes are first mentioned in the Results section, the question should be answered no.	Yes = 1 No = 0
3	Are the characteristics of the patients included in the study clearly described? In cohort studies and trials, inclusion and/or exclusion criteria should be given. In case–control studies, a case-definition and the source for controls should be given.	Yes = 1 No = 0
4	Are the interventions of interest clearly described? Treatments and placebo (where relevant) that are to be compared should be clearly described.	Yes = 1 No = 0
5	Are the distributions of principal confounders in each group of subjects to be compared clearly described? A list of principal confounders is provided.	Yes = 2 Partially = 1 No = 0
6	Are the main findings of the study clearly described? Simple outcome data (including denominators and numerators) should be reported for all major findings so that the reader can check the major analyses and conclusions. (This question does not cover statistical tests which are considered below.)	Yes = 1 No = 0
7	Does the study provide estimates of the random variability in the data for the main outcomes? In non-normally distributed data, the inter-quartile range of results should be reported. In normally distributed data the standard error, standard deviation or confidence intervals should be reported. If the distribution of the data is not described, it must be assumed that the estimates used were appropriate and the question should be answered yes.	Yes = 1 No = 0
8^a^	Have all important adverse events that may be a consequence of the intervention been reported? This should be answered yes if the study demonstrates that there was a comprehensive attempt to measure adverse events. (A list of possible adverse events is provided.)	Yes = 1 No = 0
9	Have the characteristics of patients lost to follow-up been described? This should be answered yes where there were no losses to follow-up or where losses to follow-up were so small that findings would be unaffected by their inclusion. This should be answered no where a study does not report the number of patients lost to follow-up.	Yes = 1 No = 0
10	Have actual probability values been reported (e.g., .035 rather than <.05) for the main outcomes except where the probability value is <.001?	Yes = 1 No = 0
External validity		
11	Were the subjects asked to participate in the study representative of the entire population from which they were recruited? The study must identify the source population for patients and describe how the patients were selected. Patients would be representative if they comprised the entire source population, an unselected sample of consecutive patients, or a random sample. Random sampling is only feasible where a list of all members of the relevant population exists. Where a study does not report the proportion of the source population from which the patients are derived, the question should be answered as unable to determine.	Yes = 1 No = 0 Unable to determine = 0
12	Were those subjects who were prepared to participate representative of the entire population from which they were recruited? The proportion of those asked who agreed should be stated. Validation that the sample was representative would include demonstrating that the distribution of the main confounding factors was the same in the study sample and the source population.	Yes = 1 No = 0 Unable to determine = 0
13	Were the staff, places, and facilities where the patients were treated, representative of the treatment the majority of patients receive? For the question to be answered yes, the study should demonstrate that the intervention was representative of that in use in the source population. The question should be answered no if, for example, the intervention was undertaken in a specialist center unrepresentative of the hospitals most of the source population would attend.	Yes = 1 No = 0 Unable to determine = 0
Internal validity bias		
14	Was an attempt made to blind study subjects to the intervention they have received? For studies where the patients would have no way of knowing which intervention they received, this should be answered yes.	Yes = 1 No = 0 Unable to determine = 0
15	Was an attempt made to blind those measuring the main outcomes of the intervention?	Yes = 1 No = 0 Unable to determine = 0
16	If any of the results of the study were based on “data dredging”, was this made clear? Any analyses that had not been planned at the outset of the study should be clearly indicated. If no retrospective unplanned subgroup analyses were reported, then answer yes.	Yes = 1 No = 0 Unable to determine = 0
17	In trials and cohort studies, do the analyses adjust for different lengths of follow-up of patients, or in case–control studies, is the time period between the intervention and outcome the same for cases and controls? Where follow-up was the same for all study patients the answer should yes. If different lengths of follow-up were adjusted for by, for example, survival analysis the answer should be yes. Studies where differences in follow-up are ignored should be answered no.	Yes = 1 No = 0 Unable to determine = 0
18	Were the statistical tests used to assess the main outcomes appropriate? The statistical techniques used must be appropriate to the data. For example, nonparametric methods should be used for small sample sizes. Where little statistical analysis has been undertaken but where there is no evidence of bias, the question should be answered yes. If the distribution of the data (normal or not) is not described, it must be assumed that the estimates used were appropriate and the question should be answered yes.	Yes = 1 No = 0 Unable to determine = 0
19	Was compliance with the intervention/s reliable? Where there was noncompliance with the allocated treatment or where there was contamination of one group, the question should be answered no. For studies where the effect of any misclassification was likely to bias any association to the null, the question should be answered yes.	Yes = 1 No = 0 Unable to determine = 0
20	Were the main outcome measures used accurate (valid and reliable)? For studies where the outcome measures are clearly described, the question should be answered yes. For studies which refer to other work or that demonstrates the outcome measures are accurate, the question should be answered as yes.	Yes = 1 No = 0 Unable to determine = 0
Internal validity- confounding (selection bias)		
21	Were the patients in different intervention groups (trials and cohort studies) or were the cases and controls (case–control studies) recruited from the same population? For example, patients for all comparison groups should be selected from the same hospital. The question should be answered unable to determine for cohort and case–control studies where there is no information concerning the source of patients included in the study.	Yes = 1 No = 0 Unable to determine = 0
22	Were study subjects in different intervention groups (trials and cohort studies) or were the cases and controls (case–control studies) recruited over the same period of time? For a study which does not specify the time period over which patients were recruited, the question should be answered as unable to determine.	Yes = 1 No = 0 Unable to determine = 0
23	Were study subjects randomized to intervention groups? Studies which state that subjects were randomized should be answered yes except where method of randomization would not ensure random allocation. For example, alternate allocation would score no because it is predictable.	Yes = 1 No = 0 Unable to determine = 0
24	Was the randomized intervention assignment concealed from both patients and health care staff until recruitment was complete and irrevocable? All nonrandomized studies should be answered no. If assignment was concealed from patients but not from staff, it should be answered no.	Yes = 1 No = 0 Unable to determine = 0
25	Was there adequate adjustment for confounding in the analyses from which the main findings were drawn? This question should be answered no for trials if: the main conclusions of the study were based on analyses of treatment rather than intention to treat; the distribution of known confounders in the different treatment groups was not described; or the distribution of known confounders differed between the treatment groups but was not taken into account in the analyses. In nonrandomized studies if the effect of the main confounders was not investigated or confounding was demonstrated but no adjustment was made in the final analyses the question should be answered as no.	Yes = 1 No = 0 Unable to determine = 0
26	Were losses of patients to follow-up taken into account? If the numbers of patients lost to follow-up are not reported, the question should be answered as unable to determine. If the proportion lost to follow-up was too small to affect the main findings, the question should be answered yes.	Yes = 1 No = 0 Unable to determine = 0
Power		
27^b^	Did the study have sufficient power to detect a clinically important effect where the probability value for a difference being due to chance is <5%? Sample sizes have been calculated to detect a difference of x% and y%.	Yes = 1 No = 0 Unable to determine = 0

a Item has been omitted.

b Item has been modified.

c Total core is a percentage of the sum of points accumulated.
